# Editorial: Influence of the immune system on bone metabolism

**DOI:** 10.3389/fendo.2023.1286540

**Published:** 2023-09-20

**Authors:** Alexey Sarapultsev, Bo Shuai, Desheng Hu

**Affiliations:** ^1^ Laboratory of Immunopathophysiology, Institute of Immunology and Physiology (RAS), Yekaterinburg, Russia; ^2^ Russian-Chinese Education and Research Center of System Pathology, South Ural State University, Chelyabinsk, Russia; ^3^ Department of Integrated Traditional Chinese and Western Medicine, Wuhan Union Hospital, Tongji Medical College, Huazhong University of Science and Technology, Wuhan, China

**Keywords:** bone metabolism, endocrinology, immune system, osteoporosis, therapeutic targets, bioinformatics, necroptosis

The nexus between immunology and bone metabolism has become increasingly prominent, particularly in light of a globally aging population and the increasing prevalence of bone metabolic disorders. Novel therapeutic avenues are imperative and the potential for groundbreaking interventions appears promising, especially as the molecular intricacies linking the immune system and bone metabolism become more apparent. This editorial aims to offer a concise yet comprehensive review of the key research in the field, describing the pathophysiological mechanisms and therapeutic prospects.

Hu et al. make a seminal contribution by identifying necroptosis as a critical cellular process in osteoporosis. Their findings shed light on the molecular orchestrations—RIPK1, RIPK3, and MLKL—responsible for bone microstructure degradation. Thus, their work opens the door to therapeutics targeting necroptosis to mitigate osteoporosis.

Kaur et al. employ a humanized-BLT mouse model to offer key insights into osteonecrosis of the jaw (ONJ). Their findings reveal that IFN-γ modulation via zoledronic acid and denosumab significantly impacts bone metabolism. This adds a layer of complexity to our understanding of the crosstalk between the immune response and bone metabolic disorders.

Taking advantage of technological advancements, Hao et al. adopt bioinformatics and machine learning methodologies to pinpoint immune genes vital for bone mineral density (BMD). This research not only serves as a predictive model for osteoporosis but also exemplifies how computational approaches can accelerate discovery in the field.

Lin et al. extend the discussion by examining the impact of leptin and melatonin on bone metabolism in ovariectomized rodents (OVX). Their work indicates that these compounds could serve as potential therapeutic agents for improving bone microstructure and growth.

Lastly, Fan et al. delve into the role of azilsartan in inhibiting inflammation-triggered bone resorption and osteoclastogenesis. Their research elucidates the inhibitory effect of azilsartan on RANKL-mediated osteoclastogenesis, hence suggesting a new pharmacological intervention.

Despite these seminal contributions, there are still unanswered questions that warrant attention. Understanding the cellular and molecular mechanisms by which immune cells affect bone metabolism remains an open area of investigation. Similarly, translating these molecular insights into clinical applications is another significant challenge that researchers must grapple with in the coming years.

In summary, the intricate relationship between the immune system and bone metabolism holds the key to unlocking new therapeutic pathways. As we continue to deepen our understanding through interdisciplinary research, the potential to mitigate the global health burden of bone metabolic disorders becomes increasingly realistic.

## Author contributions

AS: Conceptualization, Writing – original draft, Writing – review & editing. BS: Conceptualization, Writing – original draft, Writing – review & editing. DH: Conceptualization, Writing – original draft, Writing – review & editing.

